# PROD-ALERT 2: replicating and extending psychiatric restraint open data analysis using logarithmic estimates of reporting trends

**DOI:** 10.3389/fpsyt.2024.1325142

**Published:** 2024-07-05

**Authors:** Keith S. Reid, Owen Price

**Affiliations:** ^1^ Positive and Safe Care Team, Cumbria Northumberland Tyne and Wear NHS Foundation Trust, Newcastle upon Tyne, United Kingdom; ^2^ Faculty of Health and Life Sciences, Northumbria University, Newcastle upon Tyne, United Kingdom; ^3^ Division of Nursing & Midwifery, The University of Manchester, Manchester, United Kingdom

**Keywords:** restraint, open data, methodology, human rights, candour, information, Seni, *L*-test

## Abstract

Care, management, and statute each mandate restraint-reporting in psychiatric settings in England. PROD-ALERT in this journal (“PA1”) correlated log incidence of restraint, log institutional size, and log detention. The period was September 2020 to August 2021. It showed a clear trend among reporters. Restraint correlated with institutional size and use of legal detention. Some large detaining providers reported no restraints per month despite that trend. Inference from size suggested that non-complete reporters restrained 1,774 people per month. This paper “PA2” develops analysis repeating it for September 2021 to August 2022. PA2 shows how to count *L*-information, i.e., questionable information, added by null reports, by applying an *L*-test to data sets. PA2 uses illustrative vignettes about human height to ground *L*-information scores from English restraint reporting. In PA2, reported restraint again correlates with size and detention as in PA1. PA2 shows evolving data. Providers still follow a trend in restraint by size and detention. Providers which newly report restraint are on trend. Inference suggests that non-complete reporters restrained 1,305 people per month (536–3233), 95% CI, a large but reduced number since PA1. English restraint data have an *L*-test *L*-information score of increase in information by a factor of *L* = 145. This is as surprising as claiming that an average English man of 1.72 m is 2.64 m tall. Persons restrained per month is a robust measure continuing to log-correlate with size and legal compulsion. Providers over a certain size who report null restraint probably have some. Restraint remains underreported in England. Imputation of incomplete reporters shows a large shrinking cohort of patients detained by incomplete reporters. Knowledge of this may promote reporting. Improved reporting, and the infrastructure and integrity it demands, may help providers measure and reduce restraint. PA1 remains unrefuted. *L*-test can measure *L*-information in intuitively representable ways. The informational effect of nulls on the reliable data set is similar to a claim that an average-heighted man is as tall as people with clinical gigantism.

## 1 Introduction

### 1.1 Importance of reporting

As stated in our first paper PA1, questionable null reports are problematic (1). English mental health providers wishing to reduce incidents should report them (2). In England, providers must produce reports internally or risk sanction (3). They must publish data openly according to “Seni’s Law” (4).

### 1.2 Rates of restraint

There is an established and growing literature on quantifying restraint since at least the turn of the century (5). In Germany, per provider, detention rates correlate with restraint (6). That refuted a claim of highly-restraining teams—“our patients are different”. Flammer’s measure of restraint patient implies that larger services may have monotonically larger absolute restraint. A recent review of international reporting belies a variable picture (7).

### 1.3 Methods of PROD-ALERT 1

The PA1 correlated logarithm of size against logarithms of detention and restraint in monthly mental health metrics, the “MHMDS”, published by an English state body. Data for September 2021 to August 2022, self-reported monthly by institutions, were available as open data. They are monthly, complex, and detailed. MHS24 “Bed Days” counts size. MHS09 counts people involuntarily detained. In England, detention should be considered following restraint. MHS76 counts people subject to at least one restraint. Some institutions reported restraint at least 11 months a year, categorised as “complete” reporters. Other reporting styles are explained in the methods. We offer cleaned data (8). The present report intends to show applicability and utility of the *L*-test by adding it to PA1’s continued general description of reporting.

## 2 Methods

### 2.1 Choice of comparisons

The three metrics were chosen to give independence. They are counted, reported, and scrutinised separately. Hospitals know how many beds they have. People detained is counted by mental health act compliance officers and scrutinised by regulator the Care Quality commission (“CQC”). Restraint is reported by the restraining nursing team. Restraint can be underestimated due to loose definition, conflating successive restraints, ignoring certain postures, or minimisation. Reporting of individual restraints is hard to check with patients. A patient may know that they were restrained in a given month but not the count.

### 2.2 Funnel plotting

Monthly reports support statistical inference. Firstly it allows estimation of ratios of restraint vs. size or detention, the slope of the graph 1c and 2c. This is implicitly estimated every month. Repeated estimates allow consideration of variance. Effect size and variance allow funnel plotting. In PA1, the funnel plot for restraint vs. size was markedly asymmetric, as discussed there. Briefly, it was conjectured that highly restraining variable reporters close; reports do not often overestimate.

### 2.3 *L*-Test of *L*-information

Since PA1, another peer-reviewed method has been developed, *L*-test (9). It derives from information theory (10). Non-random data such as restraint data have a general trend and reports which questionably diverge from the trend add noise. *L*-test measures additional noise. As readers may see from Equation 1, the quantity *L* is a ratio, having no units.

For the purpose of PA2 slope estimates which are reliable are identified, drawn blue. All blue dots have *x, y* coordinates [(8.94,2.82),(9.98,3.86),…(14.6,7.05)](3sf). They estimate a slope of *y/x*. Black dots are large null reporters coordinates *x, y* [(7.14,0.0),(7.77,0.0),…(13.8,0.0)](3sf), estimating slope 0.

Blue reliable estimates are divided into two halves, *O* and *E*. Those are odd and even halves of the blue cluster of dots going out from the origin in a straight line. Those two halves of one group have a high probability of being from the same data set. This can be measured using an appropriate statistical test. *L*-test uses Mann–Whitney U. The probability will approach *p* ∼ 1.0 for a perfect trend. As the probability that *O* is like *E* (*E* ∼ *O*) is near 1.0, intuitively, we gain little new information from *O* if we know *E*. When the black dots *N* for null are joined to *O*, this makes a new group *NO* which can be compared with *E*. *E* and *NO* now have a measurably lower probability of being from the same data set. The commensurate surprise or new information occasioned is higher.

### 2.4 Features of *L*-test

Here are the procedure and equation, Equation 1.

Procedure:

1. Split complete report estimates into alternate halves even “E” and “O” odd. Call null or incomplete reports “N”.
2. Derive a probability $p\left(E∼O\right)$ that E and O are similar using the Mann–Whitney U test, approaching $p\left(E∼O\right)\;=\;1.0$ for large similar E and O.
3. Calculate $h\left(E∼O\right)$ information as $-log_{2}\left(p\left(E∼O\right)\right)$ , approaching zero as *O* and *E* seem unsurprisingly similar.
4. Construct a noisy-odd group “*NO*” the union of *N* and *O*.
5. Calculate $h\left(E∼NO\right)$ information, approaching high values as incomplete reporters make E seem questionably surprising.

6. L is the proportional increase in $h\left(E∼O\right)$ due to noise: $h\left(E∼NO\right)–h\left(E∼O\right)$


(1) $$L=\frac{h(E∼NO)–h(E∼O)}{h(E∼O)}$$

### 2.5 Demonstrating *L*-scores using stories

*L*-test quantifies *L*-information. It can be used to generate stories of comparable *L*-information for demonstration purposes, e.g., involving human height. The author Pratchett imagined an ethnic group Zoons, unable to lie (11). The developed small stories for training such as “my grandfather is quite tall”. To say one’s average grandfather is very tall is more questionable than quite tall.

### 2.6 Data sources

No institutions were excluded. NHS Digital round figures. Reports of [“26,21,11,6,1,0,missing”] are suppressed to: [“25,20,10,5,*,*,*”]. Asterisk * means ≤ 5. For the rest of the current paper, “null” signifies “*”.

### 2.7 Definitions

**Figure 1:**
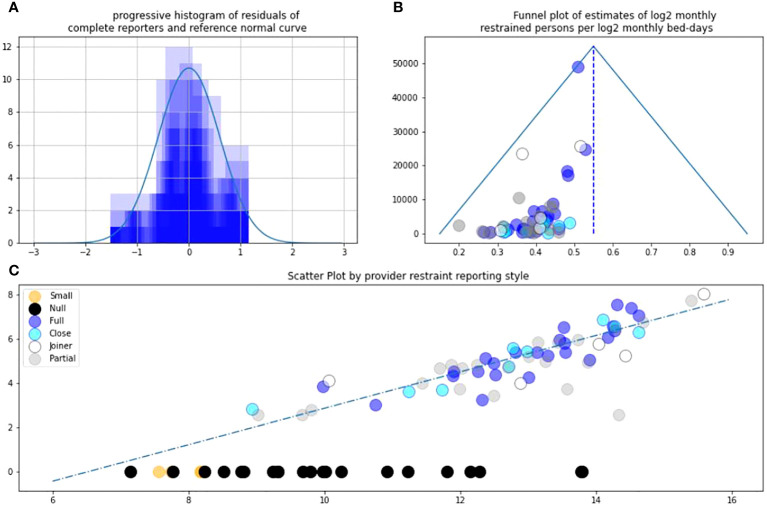
**(A)** Residuals against trend LnRestraint/LnBeds. **(B)** Asymmetric funnel plot. **(C)** L-sign scatter plot of LnRestraint/LnBeds.

Institutions were categorised using the taxonomy of PA1. As in PA1 and by definition, “joiners” reported null restraint then some restraint represented by white circles. Small providers were small enough to have null reports and other small reports such as 5 shown as orange circles. “Partial” reporters had intermittent null and were large, i.e., not in the continuous “small” series being grey circles. “Null” reporters reported null all year but were not small being black. They cannot be funnel plotted as they have no variance. All providers have bed days. Small reporters with null restraints, and “joiner” or “partial” bed-day reports were categorised “null”. Thus, if restraint was null and bed counts were partial, we did not trust restraint counts. In PA2, “complete” reporters were divided into “full” 12/12 (dark blue) and “close” 11/12 (light blue). See **Figure 1C**.

### 2.8 Data management

The procedure is expressed in Python and Julia code shareable under MIT licence. MHMDS is cleaned in an open repository.

### 2.9 Data analyses

**Figure 2:**
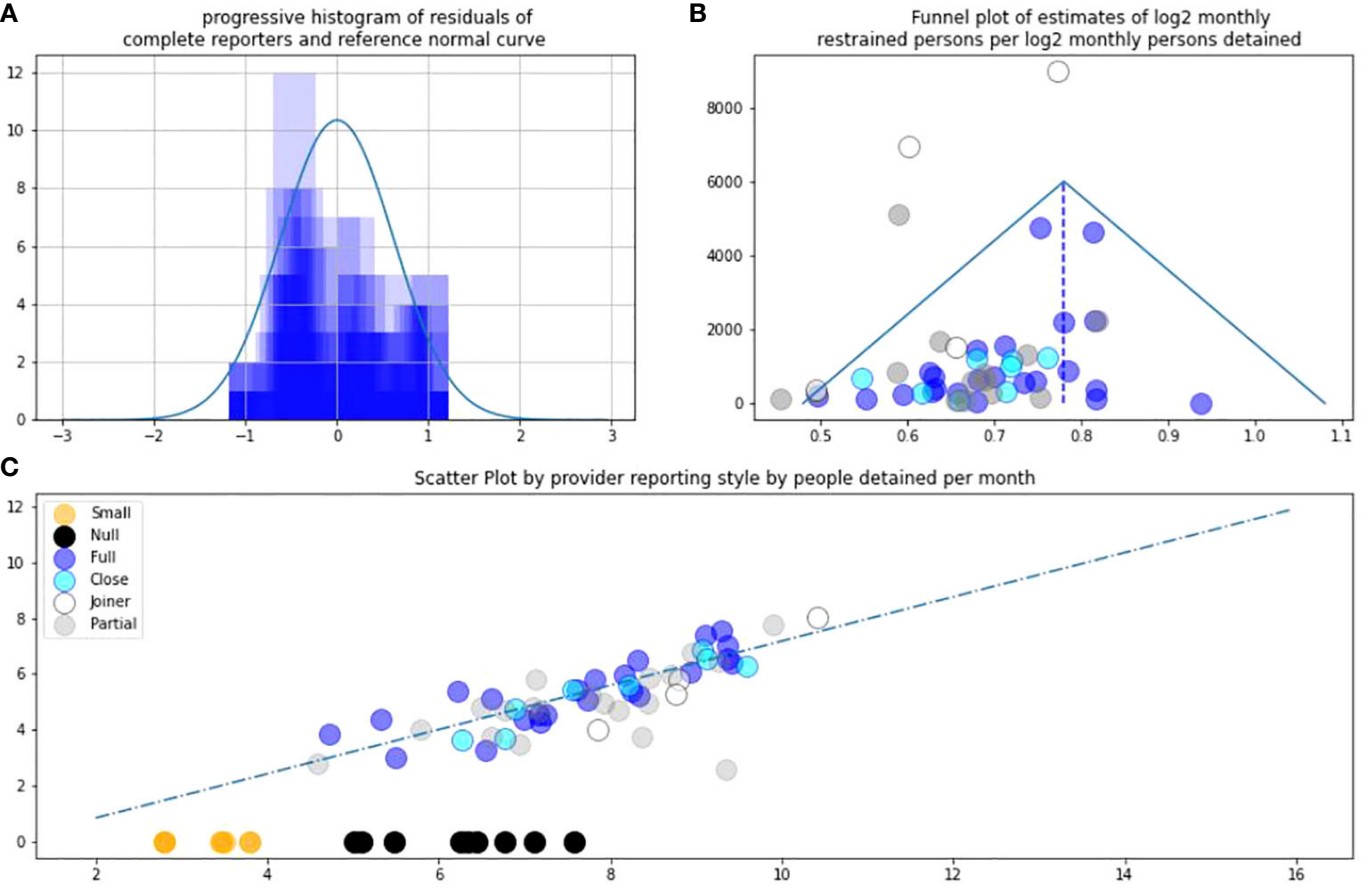
**(A)** Residuals against trend LnRestraint/LnDetention. **(B)** Asymmetric funnel plot. **(C)** L-sign scatter plot of LnRestraint/LnDetention.

Analyses were performed across two pairs of independent and dependent variable. Prior to analysis, both pairs were converted from their absolute value to their log value, using log base 2. A notional provider with approximately 4,000 beds per day has a log2 size of just under 12. Base 2 is used due to utility in information theory. Laplace correction added one to all values. The first pair were log-size and log-restraint. The second pair were log-detention and log-restraint. For size and restraint, there were a fourth and fifth analysis. The first was analysing the distribution of residuals. Log-restraint v log-size was judged to be approximately normal on visual inspection **Figure 1A**. Log-restraint v log-detention was not, as shown in **Figure 2A**. Secondly, both pairs were funnel-plotted. The x-axis position estimates “restraint/size” or “restraint/detention” by mean estimate. The inverse variance of monthly estimates from one provider is on the y-axis. Generally consistent estimates show high funnels. Thirdly, a best fit was fitted in log–log space, the estimated relationship between log-restraint and log-comparator. Fourthly, relying on normal residuals, correlation and interpolation were performed of restraint by size. That interpolated people restrained by reporters that did not completely report, i.e., white, grey, and black. Fifthly, *L*-test score was applied and illustrated.

### 2.10 Ethical permissions

No ethical permissions were needed for public data.

## 3 Results

### 3.1 General

Data comprised monthly reports from providers over a 12-month period. Providers are groups of hospitals, typically either: regional state-run quasi-autonomous “National Health Service (NHS) Trusts” or independent companies tending to be the largest providers. Providers reported data as follows for the three metrics. Due to suppression, all numeric values are multiples of 5 (see Data Sources at 2.6). Lower values are suppressed to “*”. Pragmatically for summary data, to include joiners, the final month was taken as representative.

#### 3.1.1 Persons detained per month

Reporting detained people was part of the definition of a mental health provider. That means this total group is larger and encompassed some providers who were not mental health providers. August 2022 saw 319 total entries of which 61 provided a numeric value of persons subject to detention. The range of people reported detained in the 61 numeric reports was 5–1,350, and the median (the 31st provider) was 160. The sum of the reported detained persons was 15,255. This could conceivably double-count people who moved from one provider to another.

#### 3.1.2 Patient “bed days” per month

August 2022 saw 319 entries of which 76 providers reported numeric bed-days. The range was 125–50,655, median (38th) 5,735 bed-days. Dividing by 31 days in August 4–1,634 beds, median 185. Total reported bed days was 689,430 bed-days equivalent to 22,239 beds. A sense check shows this to be larger than the detained population due to some patients being voluntary.

#### 3.1.3 Persons restrained per month

August 2022 saw 50 entries providers with 47 reported numeric figures. The range was 5–275, median (24th) 40. The sum was 2,860 persons. For clarity, the later interpolation analysis at 3.4 suggests 2,860 restrained persons to be an underestimate.

### 3.2 Size and restraint

**Figure 1A** allows correlation of complete reporter restraint vs. size because the residual approach normal distribution. **Figure 1B** shows the funnel plot remains highly asymmetric as in PA1. The blue reports are the most reliable for assessing the overall distribution. Note that incomplete reporters can have apparently high precision with this analysis if they reported a small number of similar reports in a year. **Figures 1C** and **2C** are the *L*-pattern scatter plot. Null black (and small orange) reports contribute the horizontal limb of an “L” on the scatter. The two largest providers had been nulls in PA1. In PA2, they can now be seen as one grey partial and one white joiner at the far right hand side and are welcomed. Note that they appear near the general trend.

### 3.3 Detention and restraint

As in PA1 detention provides a supportive secondary analysis. Again blue reports are the most reliable. The distribution of residuals of complete reporters appears skewed. It is skewed towards lower estimates of log restraint per log detention. The funnel plot of estimates of restraint per detention is more symmetrical than the funnel of restraint per size. See **Figure 2B**.

### 3.4 Interpolation on size

The predicted number of people restrained per month in incomplete reporters was 1,305 people per month (536–3,233) 95% CI. This is a reduction since the similar estimate in PA1 regarding the previous year.

### 3.5 Height-expressed *L*-information in the set

The *L*-test *L*-information in English restraint data is an increase of information by an increase of 145 as follows.

The probability $p\left(E∼O\right)=\;0.958$ (3sf).

The associated information is:


$$h\left(E∼O\right)\;=\;-log_{2}\left(p\left(E∼O\right)\right)=\;0.0626\;\text{bits}\;(3\text{sf}).$$

The probability $p\left(E∼NO\right)=\;0.00172$ (3sf).

The information there is: $h\left(E∼NO\right)\;=\;-log_{2}\left(p\left(E∼NO\right)\right)=\;9.19$ bits (3sf).


$$L=\frac{9.19–0.0626}{0.0626}=\frac{9.12}{0.0626}=145$$

The proportional increase is to make the information 145 times greater. A description of height of English men contemporaneous to the Pratchett novel is 1.732 m SD 6.6 cm (12). An accurate statement that a grandfather of average height is *true* and makes him equal to or taller than 0.5 of the cumulative distribution. The negative log2 of 0.5 is 1, thus carrying information 1 bit. Increasing this by 145 bits requires a probability with negative log 2 of 146, that is, 1.12 × 10^−44^ (3sf). The normal distribution is continuous extending into infinity. This allows us to estimate which height covers that percentile. It needs a height over 13.85 SD above the mean. SD is 6.6 cm; 6.6 × 13.85 = 91.41 cm, so the necessary claimed height is 264.61 cm, matching celebrities known only for their syndromic height being extremely or maximally tall.

## 4 Discussion

This study PA2 updated and extended our previous study PA1. Both studies evaluated information in mandatory restraint reporting among English mental health and related providers in consecutive years. Those years followed legislation to require reporting.

### 4.1 Limitations

These are probabilistic inferential methods. There is the chance that null reports in a given month from a large provider are true. This would entail providers restraining many people 1 month, then none for some months, or that providers who “join” had no restraints then many. This is less tenable as large providers join and report on trend, being welcomed. Limitations remain in these methods as discussed in PA1, which we summarise here. NHS Digital continue to round data for good reason. The data are incomplete, but the incompleteness is the point. Categorisations such as small, partial, joiner, may feel subjective but less subjective now that they are supported by repeat data, and have face-valid grouping, as shown in **Figure 2C**. An issue raised in PA1, of variable definitions of restraint, continues to be managed by regulators such as the CQC.

### 4.2 Interpreting trends

Like PA1, PA2 reads institution’s reports as estimates of a ratio of restraint and size plotted on a log-log graph to handle scale. As in PA1, there is a clear and believable trend. That trend is now supported, by providers who no longer report null, which is positive. It now seems reasonable to suppose that bigger providers have more restraint and that this occurs in other nations. Restraint reporting remains incomplete per the literature and publicly available documents. Consistent with PA1, our funnel and scatter plots suggest omissions of restraint reports may not be random. The most parsimonious explanation, which PA1 was reticent to state, was that some providers give incomplete reports. This is now our best explanation and appears validated in four ways, twice, using size and detention respectively each.

### 4.3 Interpreting funnels

It is intriguing that the funnel plot for restraint/detention is more symmetric than restraint/size. This importance of detention supports Flammer’s findings. Perhaps detention is a more reliable metric than size. One reason could be that detention reflects need. Perhaps detention controls for factors which lead to underestimation using size. In addition, it is possible to underestimate detention, perhaps balancing out omissions in restraint.

### 4.4 Causes of under reporting

PA1 considered causes of imprecision in restraint reporting. This brief data paper does not allow scope for policy recommendations, but a recent review of international reporting with mutual authorship does so (7). Omissions are more likely than spurious “over-reporting”. Institutions that restrain most might require to develop precision, due to scrutiny. Imprecise high restrainers might close. One cause of variance not raised on PA1 has occurred to us. Many large providers grew by merger. Perhaps they have smaller directorates or groups inside them that themselves do not report completely. Boards and directors might use our methods to predict rates of restraint in their subdivisions.

### 4.5 Utility

In PA2, in addition to the methods of PA1, the following new features or methods validate and expand our original study. Some code for analysis is open source on a repository. The remainder is available on reasonable request for replication. Importantly it can be used by regulators, media, and wider civil society, immediately. This is the intent of our sharing it. We suggest applications in sepsis reporting, drug incident reporting, and specific tertiary interventions such as seclusion. They may also be applied more broadly in any industries with safety-critical incident reporting and incomplete reports.

## Data availability statement

The original contributions presented in the study are included in the article/supplementary material. Further inquiries can be directed to the corresponding author.

## Author contributions

KR: Conceptualization, Data curation, Formal analysis, Investigation, Methodology, Software, Visualization, Writing – original draft, Writing – review & editing. OP: Writing – review & editing.
